# Clustering of 31 health conditions among multimorbid people in Fife and Tayside, Scotland: E-cohort analysis.

**DOI:** 10.23889/ijpds.v7i3.2036

**Published:** 2022-08-25

**Authors:** Adeniyi Fagbamigbe, Utkarsh Agrawal, Amaya Azcoaga-Lorenzo, Briana MacKerron, Colin McCowan

**Affiliations:** 1 University of St Andrews

## Objectives

A deeper understanding of the clustering of multimorbidity (2+) (MM) conditions by age, sex, and deprivation could help prepare health systems for adequate and effective preventive intervention. We identified the commonest conditions and their clusters contributing to MM stratified age, sex, and deprivation.

## Approach

Retrospective cohort of 431,772 patients aged >25 years, registered with a general practice in Fife/Tayside, Scotland and alive in January 2000 were followed till December 2019. We developed Multimorbidity e-Cohort for Fife/Tayside (MCFT), Scotland. MCFT combined anonymised Scottish demographic dataset, prescribing information systems records from 2009 and secondary care: hospital admission and day-case records, mental health inpatient and accident and emergency attendances from 2000 from the Scottish Safe Havens Health Informatics Centre. Elixhauser comorbidity classifications were used to identify the presence of 31 conditions. Besides descriptive statistics, dissimilarity algorithm in association rule mining techniques were used for the analysis.

## Results

33.6% and 13.3% had MM and complex MM (4+) respectively, which varied across age, sex and deprivation of the patients. The commonest condition among <50 years with MM are alcohol abuse (32%), chronic pulmonary disease and depression (24% each) compared with uncomplicated hypertension (57%), and cardiac arrhythmia (40%) among those aged 80+ years. We identified four unique clusters among multimorbid individuals (a) liver disease, alcohol, other neurological disorders and depression; (b) weight loss, Rheumatoid Arthritis/collagenm, hypothyroidism, deficiency anemia; (c) solid tumor and metastatic cancer; (4) peripheral vascular disorders, renal failure, fluid < electrolyte disorders, chronic pulmonary disease, uncomplicated hypertension/diabetes, pulmonary circulation disorders, valvular disease, congestive heart failure and cardiac arrhythmias. The clusters varied across sex, age and deprivation.

## Conclusion

Clusters of disease conditions in the MM population varied mostly by age. Alcohol misuse contributed to MM among a third of under-50. Almost half of the oldest age group have hypertension and cardiac arrythmia. When considering clustering of conditions, it is important to consider age, sex and deprivation.

